# Silver-Doped Mesoporous Calcium Phosphate for Controlled Amoxicillin Delivery and Modulation of Osteoblast-like Cell Response

**DOI:** 10.3390/pharmaceutics18070876

**Published:** 2026-07-17

**Authors:** Asmaa M. El-Tohamy, Mahmoud T. Abo-elfadl, Mostafa Mabrouk

**Affiliations:** 1 Physics Department (Biophysics), Faculty of Science, Al-Azhar University (Girls), Cairo 11884, Egypt; 2 Biochemistry Department, Biotechnology Research Institute, National Research Centre (NRC), Cairo 12611, Egypt; mt.el-fadl@nrc.sci.eg; 3 Cancer Biology and Genetics Laboratory, Centre of Excellence for Advanced Sciences, National Research Centre (NRC), Giza 12622, Egypt; 4 Refractories, Ceramics and Building Materials Department, National Research Centre (NRC), 33 El Buhooth St., Dokki, Giza 12622, Egypt

**Keywords:** mesoporous calcium phosphate, silver-doping, amoxicillin

## Abstract

**Background**: Calcium phosphate (CaP)-based materials are widely used for bone defect repair, but their clinical utility is often limited by insufficient antibacterial activity and a lack of controlled drug-release capability. To address this gap, the present study investigates whether silver doping can simultaneously enhance the microstructural properties of mesoporous CaP and modulate its capacity to deliver amoxicillin in a controlled manner, thereby combining osteoconductive, antibacterial, and antibiotic-delivery functions in a single platform. **Methods**: Mesoporous CaP was synthesized via the polymer sacrificial method and doped with silver at two concentrations (0.5 and 1.0 wt%), both with and without amoxicillin loading, to isolate the individual and combined effects of silver and antibiotic incorporation. The resulting formulations were characterized by XRD, FTIR, SEM, and BET to establish structure–property relationships linking silver content to physicochemical and microstructural features, while their functional performance was assessed through amoxicillin release in PBS over 672 h and through biocompatibility testing on MG-63 osteosarcoma cells via MTT assay at 48, 72, and 120 h. **Results**: Silver incorporation was found to improve the microstructural properties of the mesoporous CaP and to progressively reduce cumulative amoxicillin release, from approximately 45% in undoped CaP to about 20% at the highest silver content, indicating that silver doping enables tunable, sustained drug release. This modulation of release was accompanied by a favorable biological profile: at 48 h, most formulations showed only moderate effects on MG-63 viability, with comparable IC_50_ values across groups, while cytotoxicity declined and cell viability increased with longer incubation, reaching the highest proliferation at 120 h for all silver/amoxicillin-containing formulations. **Conclusions**: Together, these results demonstrate that silver doping does not compromise, and may enhance, the biocompatibility of mesoporous CaP even as it extends antibiotic release. This combination of tunable drug delivery, improved microstructure, and time-dependent biocompatibility positions silver-doped mesoporous CaP as a promising multifunctional platform for antibiotic delivery in bone regenerative medicine.

## 1. Introduction

Calcium phosphate materials have recently garnered considerable attention for their remarkable capacity to promote osteoblast differentiation into bone cells, facilitating growth on scaffold surfaces, appropriate degradation rates, and eventual complete replacement by newly formed bone tissue, while also enduring stress at the defect site [1]. Porous drug carriers may enhance the loading efficiency of antibiotic medications [2]. Owing to its chemical similarity to the inorganic phase of human bone, calcium phosphate (CaP) demonstrates remarkable biocompatibility, the capacity to sustain biological activity, and an inherent ability to promote osteogenesis [3,4,5,6,7]. Considering the importance of material properties in biomedical applications, it is crucial to understand how these characteristics influence the performance of implants within the body.

Physical performance and biological interaction influence the function of a biological implantation in the human body. The chemical, physical, and mechanical features of an implant predominantly govern its interaction with the human body in terms of biocompatibility and bioactivity [8,9,10]. Besides interacting with tissue cells, implants often connect with other cells, including bacteria, which may lead to infections from multidrug-resistant bacteria. Accordingly, next-generation metallic implants must simultaneously achieve biocompatibility and bioactivity while effectively mitigating the risk of infection [11,12]. In response to this need for antimicrobial properties, various strategies have been explored to modify implant surfaces and prevent infection.

Researchers have suggested various surface antibacterial strategies to avert early implant-associated infections, including antibacterial coatings (e.g., silver, copper, or zinc-based), surface nanotopography modifications that physically disrupt bacterial membranes, and functionalization with antibiotic- or antimicrobial peptide-loaded layers that provide localized, sustained drug release [13,14]. This means that there are elements like silver, copper or zinc that can be incorporated to interact with bacterial cell membrane causing cell death after contact. An approach involves applying a silver-infused hydroxyapatite (HA) coating that provides antibacterial characteristics while maintaining the bioactivity of the implant. Silver (Ag) is a recognized antibacterial agent effective against over 650 bacterial species, with very minimal toxicity to mammalian cells. The remarkable antibacterial capabilities of silver ions, compounds, and nanoparticles have led to their increasing use for treating infections. Previous studies indicate that silver-containing hydroxyapatite structures enhance the ability to kill bacteria, which is a positive finding for their antimicrobial properties [15,16,17]. Additionally, other studies have shown that in vivo implantation of amoxicillin-loaded biomaterials/scaffolds led to appropriate increases in bone area and thickness [18,19]. Furthermore, amoxicillin increases bone formation by inducing bone metabolism agents, such as type I collagen, Bone Morphogenic Proteins-2 (BMP-2), and Alkaline Phosphatase (ALP) [20,21]. A different investigation found that amoxicillin acquired in in vitro and in vivo studies had considerably higher BMP-2 and ALP mRNA expression [22].

Although silver has been widely explored as an antibacterial dopant for calcium phosphate-based biomaterials, most existing systems rely on dense, non-porous Ag-doped calcium phosphate or hydroxyapatite particles in which bacterial inhibition depends solely on passive ionic silver release [23,24,25,26], or on silver-doped cements and coatings evaluated against a narrow bacterial panel without pairing silver with a co-delivered, clinically established antibiotic [27,28]. Mesoporous calcium-based carriers loaded with silver have so far been directed mainly at dental disinfection or at ion-substitution-only antibacterial strategies, rather than at combining silver doping with controlled antibiotic delivery in a single mesoporous platform [29,30]. The present work addresses this gap by coupling tunable silver doping with a mesoporous calcium phosphate carrier engineered for amoxicillin release, thereby integrating two independent and potentially synergistic antibacterial mechanisms—ionic silver release and antibiotic release—within one osteoconductive system. Moreover, while previous reports have generally restricted antibacterial evaluation to Gram-positive and Gram-negative bacteria [23,27,31], the present study extends testing to a pathogenic yeast *Candida albicans*, broadening the assessed antimicrobial spectrum beyond what is typically reported for silver-doped CaP systems, while also confirming biocompatibility on osteoblast-like MG63 cells.

Building upon these promising findings, the following study explores a novel approach combining silver doping with a mesoporous calcium phosphate system for targeted drug delivery and enhanced antimicrobial activity. In this work, the amoxicillin drug was delivered via a mesoporous CaP delivery system doped with different concentrations of silver. The produced nanosystems were characterized using scanning electron microscopy (SEM), transmission electron microscopy (TEM), X-ray diffraction (XRD), and Fourier transform infrared spectroscopy (FTIR). Furthermore, the release profile was measured in phosphate-buffered saline (PBS), and the data were fitted against mathematical models to predict its kinetics. Also, the inhibition zone test was used to determine how well the system fights against Gram-positive bacteria *Staphylococcus aureus*, Gram-negative bacteria *Escherichia coli*, and harmful yeast *Candida albicans*. Finally, the biocompatibility was evaluated in vitro using human MG63 osteosarcoma cells.

## 2. Materials and Methods

### 2.1. Materials

Calcium nitrate (Ca(NO_3_)_2_.4H_2_O, 236.15 g/mol, Advent, Mumbai, India), triethyl phosphate ((C_2_H_5_O)_3_P(O), 99%, Aldrich chemical company, St. Louis, MO, USA), silver nitrate (AgNO_3_, 169.87 g/mol, Sisco research laboratories, Mumbai, India). Polyacrylamide (C_3_H_5_NO)n, average molecular weight Mn 150,000 g/mol, CAS Number: 9003-05-8, Sigma and International Chemistry Co. Ltd., Johannesburg, South Africa.

### 2.2. Mesoporous CaP Synthesis Method

A dispersion polymer solution was initially generated by dissolving 1 g of polyacrylamide in 1400 mL of distilled water at 80 °C for 2 h under continuous stirring at 1000 rpm. In this system, polyacrylamide acts as a sacrificial templating agent: its long hydrophilic chains form an entangled, hydrogen-bonded polymeric network in solution that provides nucleation sites for calcium and phosphate precursor ions and physically constrains their growth, thereby controlling the spatial distribution and interconnectivity of the resulting mineral phase. Subsequently, 0.056 mol (13.22 g) of calcium nitrate tetrahydrate was solubilized in the dispersion polymer solution, followed by the addition of 0.033 mol (6 mL) of triethyl phosphate (TEP). As these precursors dissolve, Ca^2+^ and phosphate-derived ionic species electrostatically associate with the polar amide groups of the polyacrylamide chains, nucleating an amorphous calcium phosphate phase along the polymer network rather than in free solution. This templated nucleation confines mineral growth to the interstitial spaces of the polymer matrix, establishing the precursor framework for the mesostructure ultimately obtained after polymer removal. The pH of the entire mixture was adjusted to 9.5 using ammonia solution and maintained at 80 °C under stirring for 3 h to promote controlled hydrolysis and condensation of the phosphate precursor and to favor precipitation of a calcium phosphate phase intimately intermixed with the polymer network. The resulting mixture was dried at 80 °C for 24 h to remove residual water and consolidate the polymer–mineral composite into a xerogel prior to thermal treatment. The dehydrated gel then underwent thermal treatment at 800 °C for 3 h, with a ramping rate of 5 °C/min. During heating, the polyacrylamide network undergoes progressive thermal decomposition and combustion, generating volatile by-products that diffuse out of the compact and leave behind a network of voids corresponding to the space previously occupied by the polymer chains. Because the mineral phase had nucleated along and within this polymer network during synthesis, its removal yields an interconnected mesoporous architecture whose pore size and distribution are governed by the original polymer chain conformation and packing density, rather than by post-synthesis pore-forming steps. This heat treatment is therefore a well-established method for removing the polymer template while simultaneously crystallizing the calcium phosphate phase, yielding a mesoporous structure. Because the polymer concentration, precursor stoichiometry, stirring rate, pH, and heat-treatment profile were fixed across batches, the resulting pore architecture was reproducible between synthesis runs. To incorporate silver into the calcium phosphate samples, silver nitrate was added to the above solution after the addition of TEP, at two concentrations (0.5 and 1.0 wt%). Silver ions are similarly retained within the polymer-templated precursor network prior to calcination, such that following polymer burnout, silver becomes incorporated into or onto the calcium phosphate mesostructure rather than existing as a separate phase.

### 2.3. Characterization of Mesoporous CaP

The different crystal forms in Ag-doped CaP powder samples (as prepared without further powdering) were found using an X-ray diffractometer (X-ray diffraction (XRD) model BRUKER axs, D8 ADVANCE, Karlsruhe, Germany) and in comparison to the standard CaP sample. XRD patterns were recorded over a 2θ range of 2–80° using Cu-Kα radiation (λ = 1.5406 Å) with a nickel filter, operating at 40 kV and 40 mA. Phase identification was performed by matching the recorded diffraction patterns against reference cards in the JCPDS powder diffraction database. FTIR spectra of the synthesized samples were obtained using a Fourier-transform infrared spectrophotometer (model FT/IR-6100 type A). The spectra were acquired throughout the wavelength range of 4000 to 500 cm^−1^, achieving a resolution of 2 cm^−1^. Transmission electron microscopy (TEM) (JEOL, Tokyo, Japan; JEM2100, electron microscope, and TEM) was employed to investigate morphology and particle size diameters. The Brunauer–Emmett–Teller (BET) method was employed to determine the specific surface area of each sample under varying conditions, utilizing adsorption isotherm data and the Quantachrome Nova Automated Gas Sorption System Version 1.12. It typically operates by utilizing the BET method for relative pressure (P/P_0_). We determined the pore size distribution employing a cylindrical pore model and the Barrett–Joyner–Halenda (BJH) desorption technique. The initial composition of the precursors is presented in Table 1.

### 2.4. Drug Loading, Release and Kinetics

#### 2.4.1. Pharmaceutical Loading

The efficacy and loading of amoxicillin in silver-free mesoporous calcium phosphate-doped nanopowders were assessed. Amoxicillin, an antibiotic sourced from EIPICO (Egyptian International Pharmaceutical Industries Company, Cairo, Egypt), was employed in the current research due to its substantial antibacterial effectiveness, which was the rationale for its selection. Approximately 0.1 g of mesoporous nanomaterials was submerged in 50 mL of PBS containing 0.1 g of amoxicillin powder, resulting in a final concentration of 1 mg/mL. A shaker incubator was employed at 200 rpm for 18 h to provide uniform drug loading into the mesoporous CaP structure; subsequently, the powders were dried at 37 °C.

#### 2.4.2. In Vitro Drug Release

Amoxicillin-loaded mesoporous CaP-doped and silver-free nanopowders (100 mg) dried samples were combined with 1 mL of phosphate-buffered saline (PBS) in a dialysis bag, and the drug-loaded samples were submerged in 50 cc of PBS at pH 7.4 and 37 °C. Subsequently, 3 mL of the solution was removed and replaced with 3 mL of fresh PBS at specified intervals (short immersion durations: 2, 4, 6, and 24 h; long immersion durations: 3, 5, 7, 14, 21, and 28 days). The obtained solutions were frozen for further drug concentration testing at −20 °C. The quantity of amoxicillin released into the solution was determined using a UV spectrophotometer set to a wavelength of 275 nm.

### 2.5. Kinetic Release of Amoxicillin

The performance properties of Amoxicillin were compared to mathematical models using the following equations.

Zero-order model:

(1) F = K_0_t

where *F* is the fraction of drug released at time *t*, and *K*_0_ is the zero-order release rate constant.

First-order model:

(2) ln(1 − F) = −K_1_t

where *F* is the fraction of drug released at time *t*, and *K*_1_ is the first-order release rate constant.

Higuchi model:

(3) F = K_H_t^1/2^

where *F* is the fraction of drug released at time *t*, and *K_H* is the Higuchi release rate constant.

Korsmeyer–Peppas model:

(4) F = Kt^n^

where *F* is the fraction of drug released at time *t*, *K* is the release rate constant incorporating structural and geometric characteristics of the drug delivery system, and *n* is the release exponent indicative of the drug release mechanism.

### 2.6. Antibacterial Study

Qualitative assessments were conducted in nutrient broth in accordance with [23]. The study used samples of harmful microorganisms, encompassing Gram-positive bacteria *Staphylococcus aureus*, Gram-negative bacteria *Escherichia coli*, and harmful yeast *Candida albicans*, which were made from fresh overnight broth cultures grown in nutrient broth at 37 °C [32]. The inoculum size of this pathogenic strain was prepared and calibrated to roughly 0.5 McFarland standard (1.5 × 10^8^ CFU/mL) [33]; each microorganism strain was independently inoculated with 50.0 µL into plates containing 25.0 mL of sterile nutritional agar medium (NA) [34]. Following the solidification of the medium in the 0.9 cm diameter wells of the agar plates, created previously with a 1.0 cm cork borer utilizing the well diffusion method, each well was individually filled with 100.0 µL from samples S1 (pure CaP), S2 (CaP + 0.5 wt% Ag_2_O_3_), S3 (CaP + 1 wt% Ag_2_O_3_), SD1 (CaP + amoxicillin), SD2 (CaP + 0.5 wt% Ag_2_O_3_ + amoxicillin), and SD3 (CaP + 1 wt% Ag_2_O_3_ + amoxicillin). The concentration of these samples was prepared in 100.0 mg/mL DMSO [35]. These inoculated plates were placed in the refrigerator for one hour to facilitate sample diffusion, followed by a 24 h incubation at 37 °C, with zones of inhibition (ZI) recorded in millimeters [36,37].

### 2.7. In Vitro Cell Cytotoxicity

The cytotoxic and proliferative effects of the tested samples on a human osteosarcoma cell line, MG-63, obtained from the American Type Culture Collection (ATCC), Manassas, VA, USA. The effect of the samples was measured after 48 and 72 h, and, subsequently after 5 consecutive days using the MTT assay. Dose–response curves were generated for each sample. A sub-cytotoxic dose of 50 µg/mL was further investigated for its effects on cell proliferation over 5 consecutive days. Cell morphology was examined using an inverted phase-contrast optical microscope (Olympus CKX53, Japan) at ×20 magnification. Cell viability was further assessed by means of a live/dead fluorescence staining assay, employing calcein-AM (2 µM) to label viable cells (green fluorescence) and propidium iodide (PI, 4 µM) to identify non-viable cells (red fluorescence); cells were incubated with the staining solution for 15 min at 37 °C in the dark prior to fluorescence microscopy imaging. The half-maximal inhibitory concentration (IC_50_) was determined using GraphPad Prism software version 5 (GraphPad Software Inc., La Jolla, CA, USA), and cell viability was expressed as a percentage of the untreated control.

### 2.8. Cell Death Mode

While the MTT assay quantifies overall viability, it does not reveal the mechanism of cell death. To complement these results, the mode of cell death, apoptosis versus necrosis, was evaluated using ethidium bromide/acridine orange (EB/AO) dual staining, as this distinction is relevant to biomaterial biocompatibility: apoptosis is a regulated, non-inflammatory process, whereas necrosis triggers inflammatory responses that may compromise tissue integration. MG-63 cells (1 × 10^4^ cells) were exposed to the selected undoped and silver-doped mesoporous CaP formulations loaded with amoxicillin (25 mg disc/well) for 48 and 72 h, and compared with pure amoxicillin applied to cells on standard culture plates (SPL, Pocheon, South Korea). Coverslips were rinsed with PBS and stained with EB/AO (100 µg/mL each in 100 µL PBS) for 10 min in the dark. EB/AO staining distinguishes cell states based on membrane integrity: acridine orange freely enters all cells and stains nuclei green, while ethidium bromide is excluded by intact membranes and only stains nuclei orange-red once membrane integrity is lost. Viable cells thus show uniform green nuclei; early apoptotic cells show green nuclei with condensed/fragmented chromatin; late apoptotic cells show orange-red nuclei with condensed/fragmented chromatin; and necrotic cells show uniform orange-red nuclei without chromatin condensation. Cells were imaged using a fluorescence microscope (Axio Imager Z2, Zeiss, Jena, Germany) with a fluorescence camera (×20) (AxioCam MRc3, S/N 4299, Carl Zeiss Microscopy GmbH, Jena, Germany), and images were analyzed with ZEN 2011 Blue edition software (Zeiss). Results were expressed as percentages of viable, early apoptotic, late apoptotic, and necrotic cells at each time point relative to untreated controls.

### 2.9. Statistical Analysis of Data

Data is expressed as mean ± standard deviation (SD). The dose–response curves were analyzed by non-linear regression employing five-parameter logistic curve equations, utilizing GraphPad Prism Version 10.2.0 (335) for MacOS, GraphPad Software, San Diego, CA, USA, www.graphpad.com (accessed on 25 November 2025).

## 3. Results and Discussion

### 3.1. Physicochemical Characterization

#### 3.1.1. X-Ray Diffraction Analysis

**Figure 1:**
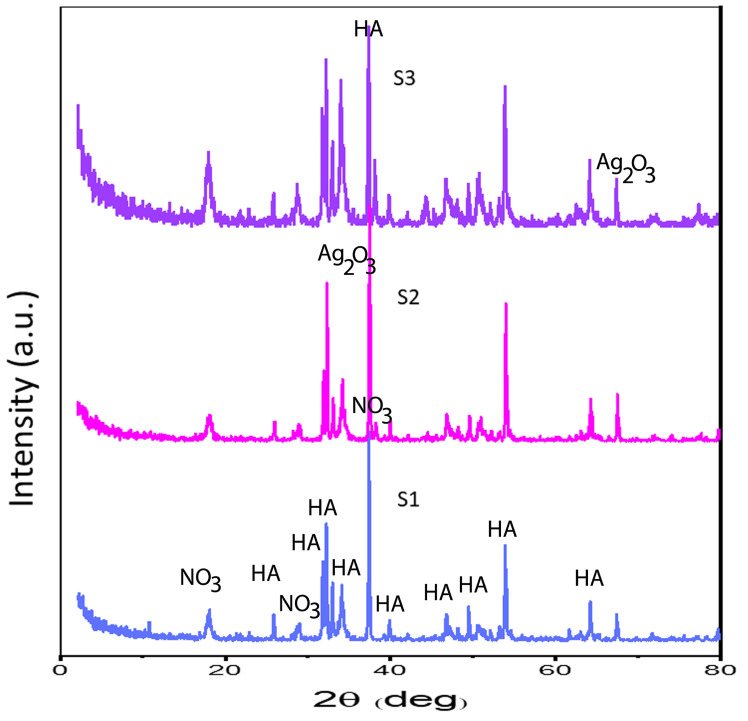
X-ray diffraction spectra of synthesized silver-doped mesoporous CaP samples compared to pure CaP.

XRD analysis was conducted on the synthesized powders to assess their crystalline structure. Figure 1 presents the diffraction patterns of pure CaP (S1), CaP doped with 0.5 wt% Ag_2_O_3_ (S2), and CaP doped with 1 wt% Ag_2_O_3_ (S3), all prepared by the sacrificial polymer template method.

In all three samples, the ten principal diffraction peaks at 2θ = 25.8°, 31.8°, 32.1°, 34.1°, 39.8°, 47°, 49.5°, 53.6°, and 64.4° are indexed to the (002), (211), (112), (300), (202), (310), (222), (213), (004), and (323) lattice planes of hexagonal hydroxyapatite (JCPDS card No. 09-0432), confirming the hydroxyapatite (HA) phase as the dominant crystalline phase across all compositions. Supplementary peaks at 2θ ≈ 17.8°, 29°, and 39.8° are attributed to residual nitrate species, the intensity of which increases with silver nitrate content, consistent with the literature [38,39]. Additional reflections at 2θ ≈ 37.5° and 67.4°, which grow progressively with Ag_2_O_3_ loading, are assigned to Ag_2_O_3_ and minor impurity phases, respectively [40,41].

Notably, the HA peak positions remain essentially unchanged across S1, S2, and S3, with no systematic shift to higher or lower 2θ values observed with increasing silver content. This absence of peak shift is mechanistically significant: it indicates that silver is not substituting for Ca^2+^ within the HA lattice, since ionic substitution would alter the unit cell dimensions and produce a measurable shift in peak position (given the larger ionic radius of Ag^+^, ~1.15 Å, relative to Ca^2+^, ~1.00 Å). This suggests that the Ag_2_O_3_ is predominantly present as a discrete secondary phase dispersed within the HA matrix, rather than substituting into the HA crystal lattice. In other words, silver doping in this system modifies the material at the level of phase composition and dispersion rather than at the level of the HA crystal lattice itself. The latter process would otherwise produce measurable peak shifts arising from unit cell expansion or contraction. The low intensity of the Ag-related reflections in S2 and S3 is consistent with the small quantities added (0.5–1 wt%) and the fine dispersion of silver oxide within the HA matrix. It should be acknowledged, however, that definitive discrimination between surface-segregated and lattice-substituted silver at these low concentrations would require Rietveld refinement analysis, which is identified as an avenue for future investigation. Overall, the XRD results confirm good crystallinity across all samples, with the HA phase well preserved upon silver doping.

#### 3.1.2. FTIR Analysis

**Figure 2:**
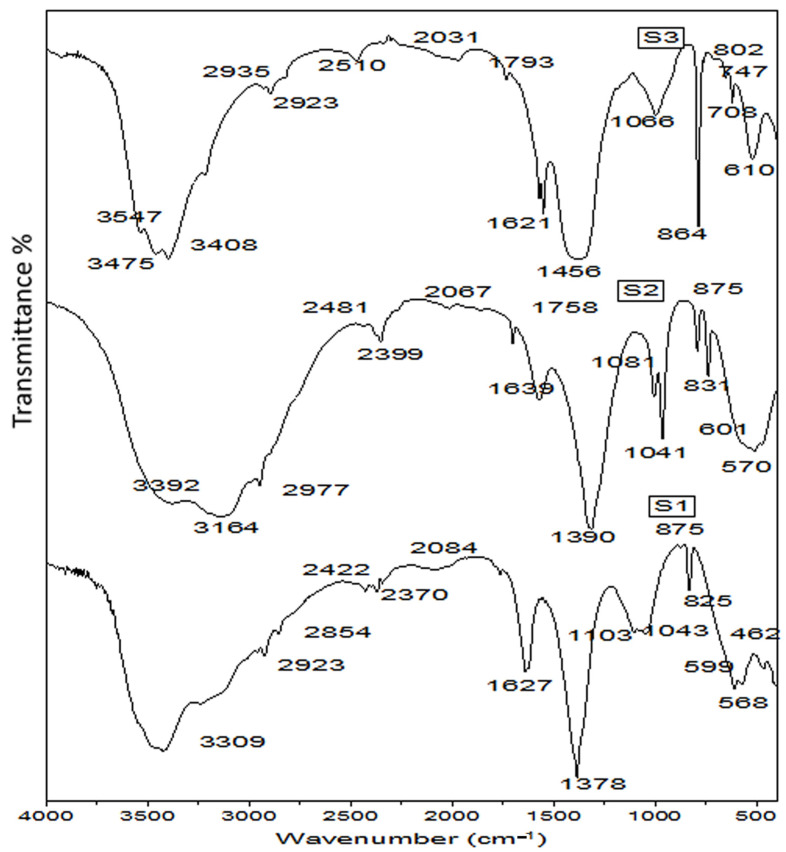
FTIR spectra of synthesized silver-doped mesoporous CaP samples compared to pure CaP.

Fourier-transform infrared spectroscopy (FTIR) was performed on the synthesized samples, and the resulting spectra are presented in Figure 2, to identify and confirm the functional groups present in the Ag_2_O_3_-doped hydroxyapatite (Ag-HA) powders.

The spectra distinctly reveal the existence of numerous vibrational modes associated with hydroxyl groups and phosphates. The broad bands observed in the 3200–3600 cm^−1^ range are associated with the H–O–H stretching vibrations of adsorbed water, while the band at around 1627 cm^−1^ is indicative of the presence of adsorbed water molecules, both of which confirm the presence of the HA phase. These bands diminished in intensity in S2 and S3 with the progressive incorporation of silver, suggesting a reduction in surface-adsorbed water content upon Ag_2_O_3_ doping. Bands at 2854 cm^−1^ and 2923 cm^−1^ are attributed to the symmetric and asymmetric C–H stretching vibrations of residual carbonaceous species originating from the sacrificial polymer template used during synthesis. The bands at 2370 cm^−1^ and 2422 cm^−1^ correspond to atmospheric CO_2_ absorption, while the band in the 1950–2100 cm^−1^ range is associated with combination modes of the [PO_4_] ν_3_ and ν_1_ vibrations, and the band at 2084 cm^−1^ is attributed to carbonate (CO_3_^2−^) species incorporated within the HA lattice, as commonly reported for biologically relevant apatites [42].

The principal functional groups characteristic of the HA structure are the phosphate and hydrogen phosphate bands. The bands at 462 cm^−1^, 568 cm^−1^, 599 cm^−1^, 1043 cm^−1^, and 1103 cm^−1^ are assigned to PO_4_^3−^ groups, corresponding to the ν_3_ and ν_4_ stretching and bending vibrations of the P–O bonds, while the band at 875 cm^−1^ is attributed to HPO_4_^2−^ ions [42]. The band at 1378 cm^−1^ is ascribed to CO_3_^2−^ groups, and the band at 820 cm^−1^ is attributed to NO_3_^−^ residues from the synthesis process, both of which are consistent with the XRD findings [43]. Collectively, the FTIR results confirm that the HA phase is the dominant component in all three samples and that silver incorporation does not alter the fundamental HA structure, in agreement with the XRD analysis. Furthermore, given that FTIR is capable of probing interactions between a drug, its carrier, and the biological environment, providing insights into drug release and stability, the presence of characteristic O–H stretching bands across all samples is particularly significant, suggesting sustained drug release potential and physicochemical stability of the system, which renders it a promising candidate for biomedical applications [44].

### 3.2. Morphological and Microstructural Properties

**Figure 3:**
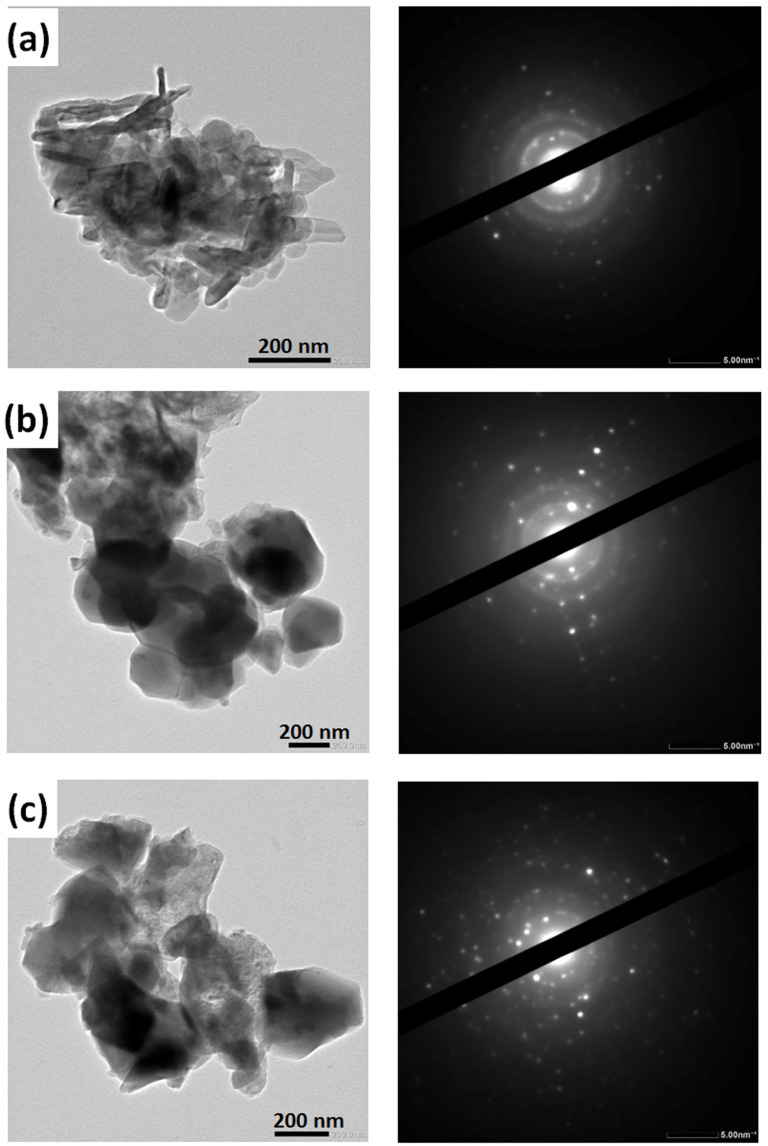
TEM for silver-doped mesoporous samples: (**a**) S1, (**b**) S2, and (**c**) S3.

TEM micrographs of samples S1, S2, and S3 are presented in Figure 3, along with their corresponding selected area electron diffraction (SAED) patterns. The diameters of the fabricated mesoporous nanoparticles fall in the following ranges: S1, approximately 100–350 nm; S2, approximately 80–300 nm; and S3, approximately 90–320 nm. Notably, sample S3 exhibits a broader and more compact aggregate.

Across the three TEM images, the Ag-doped samples (S2 and S3) (Figure 3b,c) look less plate-like and more compact/rounded than the pure CaP (S1) image (Figure 3a), with a tendency toward larger, better-defined subunits inside the agglomerates. This morphological effect is consistent with the XRD finding that silver does not enter the HA lattice: rather than altering the intrinsic crystal structure, Ag_2_O_3_ appears to act at the nucleation and growth stage, influencing particle morphology through surface and interfacial effects. In published Ag-doped calcium phosphate systems, Ag addition has been reported to change particle shape from plate-like or needle-like toward more ellipsoidal or irregular granular morphologies, along with shifts in particle size distribution. Your figure is consistent with that trend: Ag doping appears to suppress the highly flaky, loosely stacked morphology seen in pure CaP and promote denser, more rounded mesoporous nanoparticles [5]. The SAED patterns in all three panels show concentric rings with many spots, indicating nanocrystalline material made of many small randomly oriented domains rather than a single crystal. The pure sample and the Ag-doped samples all retain ring-like patterns, which suggests that Ag incorporation did not destroy the overall calcium phosphate framework, but likely modified crystallinity, domain size, or defect density. In related Ag-doped CaP and bioactive phosphate systems, Ag often does not form a strong separate crystalline phase at low/moderate loading; instead it may be incorporated substitutionally or remain below the detection threshold, while the host CaP diffractogram remains broadly similar [15]. The main Ag-doping effect visible here is morphological rather than a complete phase transformation: the particles become more compact, more rounded, and somewhat less plate-like, while the diffraction still reflects a nanocrystalline/polycrystalline calcium phosphate host. This usually points to Ag influencing nucleation and growth kinetics, likely increasing defect density and altering surface energy so that growth becomes less anisotropic [45].

### 3.3. BET Surface Area Measurements

The structural parameters derived from nitrogen adsorption analyses are summarized in Table 2. The pore radii of all samples ranged from 2 to 50 nm, classifying them as mesoporous materials according to IUPAC porosity classification criteria. Table 2 demonstrates that the specific surface area, pore volume, and pore radius decreased in the order S1 > S2 > S3, with corresponding values of 24.36, 12.28, and 10.56 m^2^/g for specific surface area, and 4.54, 3.95, and 3.37 cm^3^/g for pore volume, respectively. This progressive decline in surface area and pore volume with increasing silver content indicates that Ag_2_O_3_, present as a dispersed secondary phase rather than a lattice substituent, partially occupies or occludes pore channels and interparticle void spaces within the mesoporous framework, thereby reducing the accessible porosity without disrupting the underlying HA mesostructure itself.

**Figure 4:**
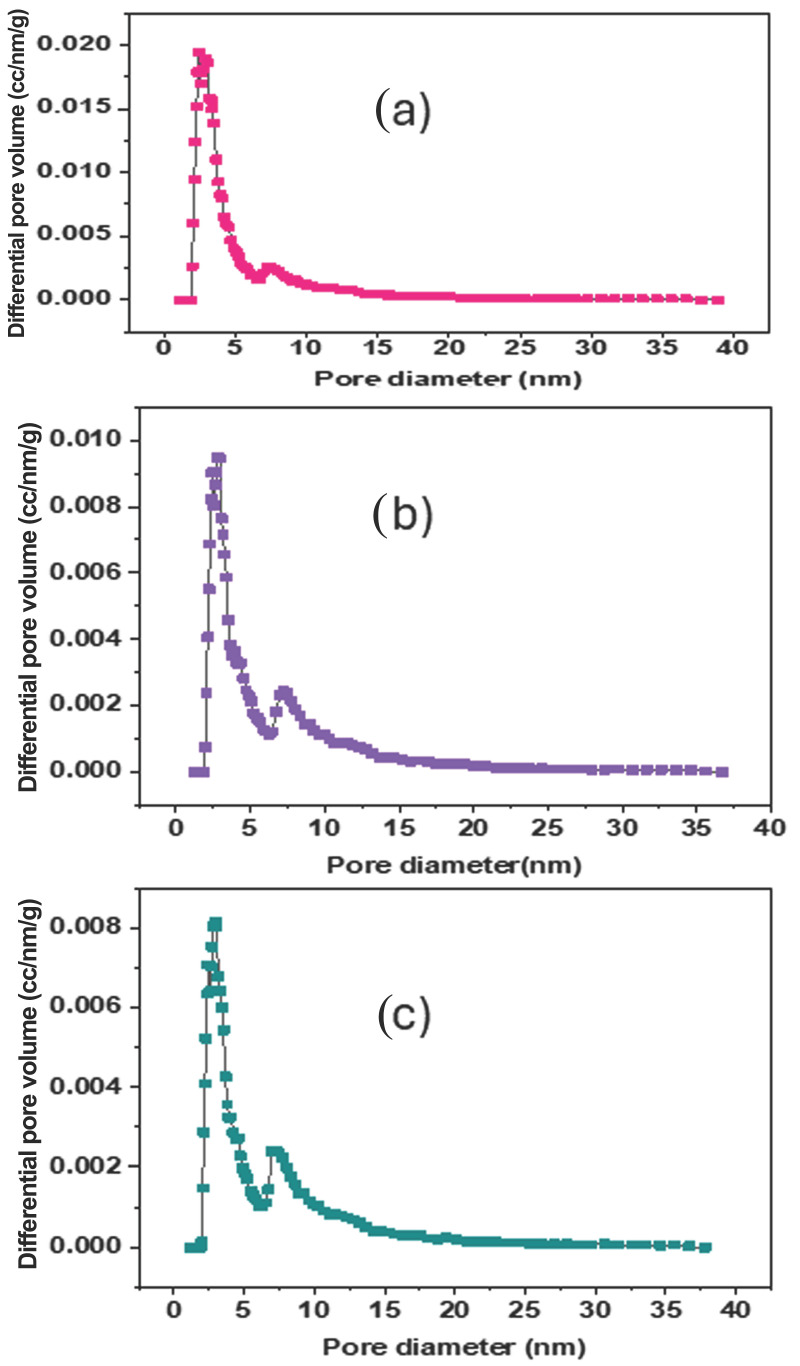
Pore size distribution of silver-doped mesoporous samples: (**a**) S1, (**b**) S2, and (**c**) S3.

In contrast, the mean pore radius followed the inverse trend S2 > S3 > S1, with values of 17.53, 14.70, and 14.61 nm, respectively. Figure 4 illustrates the pore size distribution of samples S1, S2, and S3. The mean pore radius of pure CaP (S1) is 14.61 nm, while those of the 0.5 wt% and 1.0 wt% Ag_2_O_3_-doped CaP samples (S2 and S3) are 17.53 nm and 14.70 nm, respectively, as determined by N_2_ physisorption employing the BJH method (Table 2).

**Figure 5:**
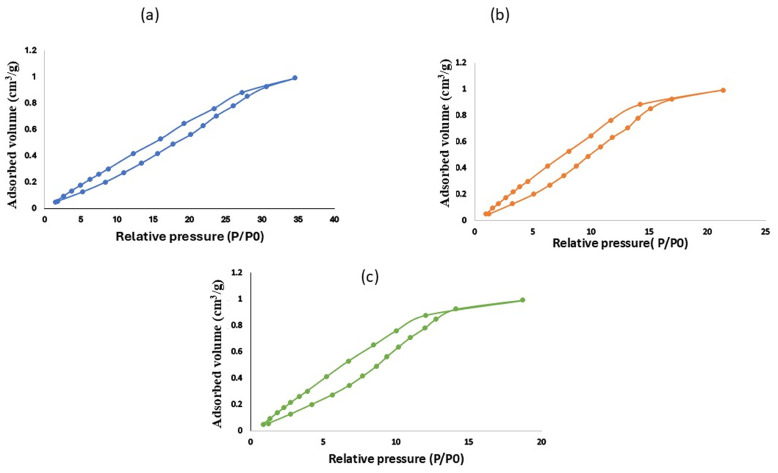
Nitrogen adsorption isotherm for the prepared samples: (**a**) S1, (**b**) S2 and (**c**) S3.

The BJH pore radius of pure CaP (S1) is 1.92 nm, whereas the corresponding values for the 0.5 wt% and 1.0 wt% Ag_2_O_3_-doped CaP counterparts are 1.91 nm and 1.92 nm, respectively (Table 2). Sample S1 exhibits a narrow pore size distribution comparable to that of S2 and S3, suggesting that the pore structure remains largely unchanged upon silver doping. Taken together with the reduction in surface area and pore volume, this indicates that silver doping narrows the population of accessible pores and blocks a fraction of the porous network, consistent with surface deposition of Ag_2_O_3_, rather than reorganizing the mesopore architecture itself. The N_2_ physisorption isotherms shown in Figure 5 indicate that all three samples exhibit Type II/IV-like isotherm behaviour with hysteresis loops, consistent with the characteristics of mesoporous materials, rather than the Type I isotherms typically characteristic of strictly microporous solids.

The progressive shift in the hysteresis loop and the reduction in adsorbed volume from S1 to S3 are consistent with the progressive decrease in surface area and pore volume associated with increasing Ag_2_O_3_ content [46].

Pore dimensions are expected to influence drug release kinetics; the relatively slower release observed may be attributed to greater drug distribution throughout the pore network, as reported in the literature [47]. These results suggest that drug release from a porous material is more facile, tunable, and rapid compared with its non-porous counterpart, an advantage attributable to the role of porosity in governing physicochemical properties of the synthesized nanopowders. As corroborated by BET surface area analysis, the porous CaP samples represent excellent candidates for drug loading applications. The elevated surface area of the CaP samples was further confirmed to augment drug release efficacy. In summary, the combined XRD, TEM, and BET data indicate that silver incorporation in this system does not proceed via substitution into the HA crystal lattice, but rather via deposition of Ag_2_O_3_ as a finely dispersed secondary phase on particle and pore surfaces. This mode of incorporation preserves the crystallographic identity and phase purity of the HA host (XRD), while producing measurable physicochemical consequences: it modifies particle morphology toward more compact, rounded structures (TEM), and reduces specific surface area and pore volume in a dose-dependent manner by partially occluding the mesoporous network (BET), without collapsing the underlying mesostructure.

### 3.4. In Vitro Drug Release

**Figure 6:**
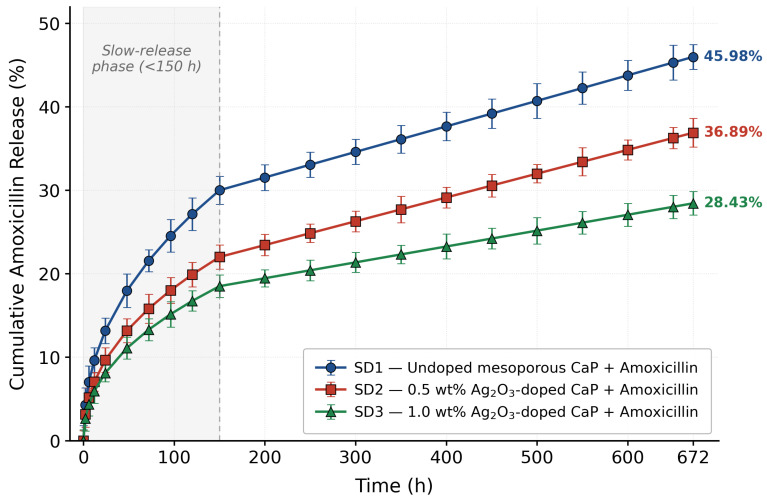
Amoxicillin cumulative release (%) in PBS.

Since the drug-loaded samples (SD1, SD2, and SD3) were prepared by complete evaporative drying of the amoxicillin loading solution in the absence of filtration or washing, the entire drug content was retained within each sample, ensuring a consistent nominal amoxicillin loading of 10 wt% across all three compositions irrespective of silver content. The nature of the interactions between the drug and its carrier, together with the microstructure of the carrier, is well established to influence drug release behaviour [48]. The drug release profiles of the three samples were examined over a period of 672 h, as shown in Figure 6.

Across all compositions, cumulative release increased gradually throughout the entire observation period. All three samples demonstrated comparable amoxicillin release behaviour over 672 h. Slow release was observed during the initial 150 h for all samples (Figure 6), during which cumulative release reached approximately 30% for SD1 (mesoporous CaP), approximately 22% for SD2 (0.5 wt% Ag_2_O_3_-doped CaP), and below 20% for SD3 (1.0 wt% Ag_2_O_3_-doped CaP), the latter being attributable to its lower specific surface area as determined by BET analysis [48]. Beyond 150 h, release profiles transitioned to a linear increase until reaching maximum cumulative releases of 45.98%, 36.89%, and 28.43% for SD1, SD2, and SD3, respectively, after 672 h. Three complementary mechanisms are proposed to account for this silver-dependent reduction in release. First, regarding pore accessibility, the BET data indicate that silver incorporation progressively reduces both specific surface area (24.36, 12.28, and 10.56 m^2^/g for S1, S2, and S3) and pore volume (4.54, 3.95, and 3.37 cm^3^/g), consistent with partial occlusion of the mesoporous network by dispersed Ag_2_O_3_. Since drug loading and release in mesoporous carriers occur predominantly through pore surfaces and channels, this reduction in accessible surface area and pore volume directly limits the amount of amoxicillin that can be loaded near the surface and readily released, and instead confines a larger proportion of the drug to less accessible internal domains. Second, regarding diffusion limitations, the reduced pore volume and partially occluded channels in SD2 and SD3 are expected to increase the tortuosity of the diffusion pathway that amoxicillin molecules must traverse to reach the release medium. Even though the mean pore radius of the Ag-doped samples (17.53 and 14.70 nm for S2 and S3) is comparable to or larger than that of undoped CaP (14.61 nm), the presence of Ag_2_O_3_ at pore walls and entrances may create localized constrictions that impede diffusive transport, so that overall release remains diffusion-limited despite the larger average pore radius. Third, regarding drug–matrix interactions, silver ions and Ag_2_O_3_ nanodomains dispersed within the CaP matrix may interact with amoxicillin through electrostatic or coordinative interactions between Ag^+^ and the amine, carboxyl, and β-lactam functional groups of the amoxicillin molecule. Such interactions could increase the affinity of amoxicillin for the silver-modified pore surfaces relative to unmodified HA, thereby slowing its desorption and diffusion into the release medium and contributing, together with the physical pore-accessibility and tortuosity effects described above, to the progressively lower cumulative release observed with increasing silver content.

The gradual in vitro degradation of the samples resulted in a progressive increase in drug release rate, consistent with a controlled release mechanism. Furthermore, the presence of silver has been reported to prolong and modulate drug release kinetics, rendering it a favourable approach in biomedical applications [49]. The combined effect of reduced pore accessibility, increased diffusional tortuosity, and possible drug–matrix interaction therefore provides a more complete explanation for the slower and lower-magnitude release from Ag_2_O_3_-doped samples than pore radius alone. The slower drug release observed in Ag_2_O_3_-doped samples may additionally be attributed to their larger mean pore radius relative to the undoped CaP sample (Table 2), which is anticipated to promote greater physical retention of amoxicillin within the pore channels, resulting in a more sustained release profile. Porous structures augment surface area, thereby expanding the available sites for cell attachment and facilitating chemical bonding with adjacent tissue [50]. Furthermore, a high degree of porosity modulates bioactivity by directly governing structural permeability, which not only regulates the initial rate of tissue regeneration but also enhances the capacity for sustained drug release as the material undergoes degradation. The degradation of a biomaterial within a biological environment constitutes one of the most critical variables governing implant performance, as this property is directly related to the duration of its retention after implantation. As evidenced by the BET data, the reduced surface areas of SD2 and SD3 relative to SD1 account for the slower drug release profiles of the silver-containing samples compared to pure CaP. All samples demonstrated effective drug delivery performance; however, selection of the optimal formulation should be guided by the mechanical and biological demands of the intended implantation site. The slower drug release exhibited by the Ag_2_O_3_-doped samples may further reflect their superior microstructural characteristics, which confer enhanced drug retention capacity relative to the undoped CaP alone.

### 3.5. Kinetics Release of Amoxicillin

**Figure 7:**
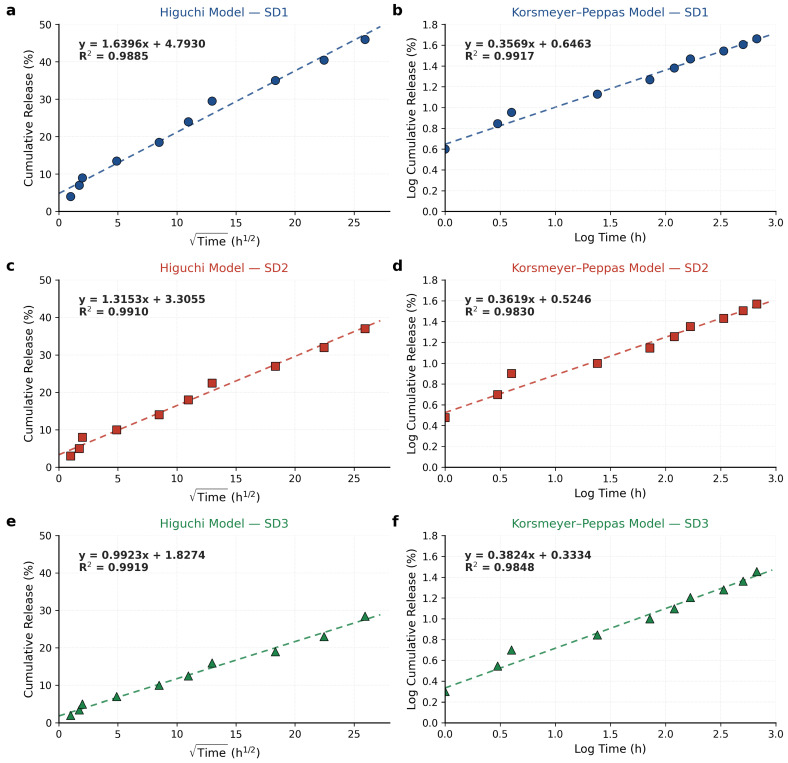
Fitting release data of samples SD1 (**a**,**b**), SD2 (**c**,**d**) and SD3 (**e**,**f**) against Huguishi and Krosmeyer–Peppas mathematical models.

The drug release characteristics were examined by fitting the experimental data to several kinetic models. Specifically, release profiles were evaluated against zero-order, first-order [51,52], Higuchi [53], and Korsmeyer–Peppas models [54,55]. Model suitability was evaluated using the coefficient of determination (R^2^). The Korsmeyer–Peppas model is expressed as F = K·t^n^, where F denotes the cumulative fraction of drug released at time t, K represents the release rate constant, and n is the release exponent governing the drug release mechanism. A value of n ≤ 0.5 indicates Fickian diffusion. Values of n between 0.5 and 1 indicate anomalous (non-Fickian) transport, encompassing both diffusion-controlled and swelling-controlled mechanisms. For n > 1, the release follows case-II transport, characterized by polymer degradation and the relaxation or expansion of polymer chains. The fitted release profiles and corresponding R^2^ values for all models are presented in Figure 7.

Based on the R^2^ values depicted in Figure 7a,b, the cumulative drug release of sample SD1 was best described by the Korsmeyer–Peppas model, indicative of coupled diffusion and erosion mechanisms, while samples SD2 and SD3 shown in Figure 7c,d and Figure 7e,f, respectively, were best described by the Higuchi model. The superior surface area, pore radius, and pore volume of the undoped CaP sample (SD1) are consistent with its distinct release mechanism relative to the silver-doped counterparts. The drug release profiles for other samples CaP doped with 0.5% Ag_2_O_3_ and CaP doped with 1.0% Ag_2_O_3_ followed Higuchi’s kinetic model, indicative of a diffusion-governed release mechanism. Linear regression analysis was applied to the release data of each sample, yielding R^2^ values that demonstrate a strong goodness-of-fit for the respective models; the corresponding parameters are summarized in Table 3.

The drug release profiles obtained from the mesoporous CaP samples demonstrate that all formulations sustained amoxicillin delivery over a period of 28 days, suggesting their potential as controlled-release platforms for amoxicillin therapy. Among the formulations, SD1 exhibited the highest cumulative drug release relative to SD2 and SD3. This discrepancy in release rates is attributed to the presence of silver in the doped formulations, which has been reported to modulate drug release characteristics [56]. Within the framework of the Higuchi model, the regression coefficients (R^2^) were 0.988, 0.991, and 0.991 for SD1, SD2, and SD3, respectively. Correspondingly, the Korsmeyer–Peppas model yielded coefficients of 0.991, 0.983, and 0.984 for SD1, SD2, and SD3, respectively. These findings suggest that the drug release kinetics from these carrier systems can be effectively described by both the Higuchi and Korsmeyer–Peppas models. The Higuchi model is particularly well-suited for characterizing the release of both water-soluble and poorly soluble drugs from a variety of matrices, as reported in the literature [57,58]. The successful application of these models indicates that the tested formulations facilitate controlled drug delivery and enable predictive modeling of drug release over extended periods, a feature of considerable importance for ensuring therapeutic efficacy. In summary, the results confirm that the investigated formulations, particularly SD1, represent promising candidates for the controlled release of amoxicillin, with prospective utility in clinical sustained-release delivery applications. It should be noted that the static PBS system used in this study does not fully replicate the in vivo environment; factors such as enzymatic degradation, dynamic fluid flow and clearance, and interactions with proteins and biological fluids may accelerate or otherwise alter the amoxicillin release profile in vivo, and further validation under physiologically relevant or in vivo conditions is warranted.

### 3.6. Antibacterial Study

**Figure 8:**
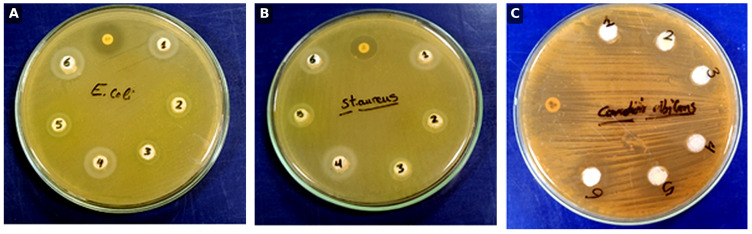
Representative agar disc-diffusion plates showing antimicrobial activity against (**A**) *Escherichia coli*, (**B**) *Staphylococcus aureus*, and (**C**) *Candida albicans*. Discs: 1 = control; 2 = S1 (undoped mesoporous CaP); 3 = S2 (0.5 wt% Ag_2_O_3_-doped CaP); 4 = S3 (1.0 wt% Ag_2_O_3_-doped CaP); 5 = SD2 (0.5 wt% Ag_2_O_3_-doped CaP + amoxicillin); and 6 = SD3 (1.0 wt% Ag_2_O_3_-doped CaP + amoxicillin).

The antibacterial activity of the samples was evaluated by measuring the zone of inhibition produced by mesoporous calcium phosphate nanopowders in their undoped form, following silver doping, and upon loading with amoxicillin. Sample discs were separately incubated with *Escherichia coli*, *Staphylococcus aureus*, and *Candida albicans*, and the results are summarized in Table 4 and illustrated in Figure 8.

As evident from Table 4 and Figure 8, pure CaP (S1) demonstrated comparatively lower antibacterial activity, exhibiting inhibition zones of 10.0 mm against *Escherichia coli*, 9.5 mm against *Staphylococcus aureus*, and 9.8 mm against *Candida albicans*. For sample S2, the zone of inhibition increased to 12.0 mm against *Escherichia coli* and 12.0 mm against *Staphylococcus aureus*, while measuring 11.0 mm against *Candida albicans*. Sample S3 exhibited enhanced antimicrobial activity, with inhibition zones of 15.0 mm against *Escherichia coli*, 15.0 mm against *Staphylococcus aureus*, and 14.0 mm against *Candida albicans*. This progressive enhancement in antimicrobial efficacy with increasing silver content is consistent with the well-documented concentration-dependent antibacterial activity of silver ions, as reported in the literature [59,60,61]. Upon incorporation of amoxicillin into the mesoporous CaP matrix of sample SD1, the inhibition zones expanded to 15.0 mm against *Escherichia coli* and 16.0 mm against *Staphylococcus aureus*, while remaining at 13.0 mm against *Candida albicans*. Formulation SD2 demonstrated further enhancement of antibacterial efficacy, particularly against *Staphylococcus aureus*, attributable to the synergistic action of silver ions and the antibiotic agent. The addition of 1 wt% Ag_2_O_3_ in combination with amoxicillin (SD3) yielded the most pronounced antimicrobial performance across all tested microorganisms. These findings are consistent with the established mechanism by which silver ions interact with bacterial cell membrane proteins, ultimately leading to cell death [62,63]. Furthermore, silver has been shown to inhibit the adhesion of microorganisms to biomaterial surfaces, thereby further augmenting its antimicrobial efficacy [64]. Against *Escherichia coli*, the most effective samples were S3, SD1, and SD3, yielding inhibition zones of 15.0 mm, 15.0 mm, and 16.5 mm, respectively. Against *Staphylococcus aureus*, samples SD1 and SD3 demonstrated the greatest efficacy, with inhibition zones of 16.0 and 17.0 mm, respectively. The pronounced antibacterial efficacy of SD1 may be attributable to the presence of calcium and hydroxyl groups, which can locally modulate environmental pH, creating conditions unfavorable for bacterial proliferation, in conjunction with the antimicrobial action of amoxicillin. Furthermore, the release of Ca^2+^ and PO_4_^3−^ ions from hydroxyapatite has been reported to contribute to the antibacterial activity of HA-based materials [65,66,67]. Against *Candida albicans*, samples S3 and SD3 exhibited the highest antifungal efficacy, with inhibition zones of 14.0 mm and 16.0 mm, respectively. The antibacterial mechanism of silver is largely attributed to its cationic nature, which confers a propensity to interact with negatively charged biomolecules, including phosphorus- and sulfur-containing constituents of cell membranes, proteins, and DNA [68,69,70,71,72,73]. The release of silver ions serves as the principal mechanism of microbial elimination. Upon contact, silver ions rapidly associate with the cell wall and cytoplasmic membrane through their strong affinity for sulfur moieties and electrostatic interactions [68,73]. Consequent disruption of the bacterial envelope increases cell permeability, ultimately culminating in cell lysis. Intracellularly, free silver ions impair respiratory enzyme function, triggering the generation of reactive oxygen species (ROS) that interfere with adenosine triphosphate (ATP) synthesis. ROS constitute the primary mediators of DNA damage and membrane disruption. Additionally, silver ions have been shown to suppress protein synthesis, occasionally resulting in ribosomal denaturation within the cytoplasm [70,71].

The combination of amoxicillin and silver demonstrated superior bactericidal efficacy relative to either agent administered individually. Moreover, concurrent administration permitted the use of reduced concentrations of both amoxicillin and silver to achieve comparable antibacterial effects [72,73]. The basis of this synergy can be attributed to several mechanistic factors. Combination therapy typically engages two agents that target distinct biological pathways, stages, or enzymes, such that bacterial resistance to one agent does not preclude the antimicrobial activity of the other via an independent, non-resistant mechanism. Furthermore, the interaction between silver and amoxicillin has been proposed to yield a novel chemical entity in which silver occupies the core and amoxicillin the outer shell; this complex adheres to the bacterial cell membrane and inflicts greater damage through the synergistic action of both components. Concurrently, amoxicillin compromises cell wall integrity, thereby facilitating the deeper penetration of silver nanoparticles into the cell and potentiating their capacity to impair DNA integrity, resulting in enhanced bactericidal activity [72,73].

### 3.7. In Vitro Cell Cytotoxicity

**Figure 9:**
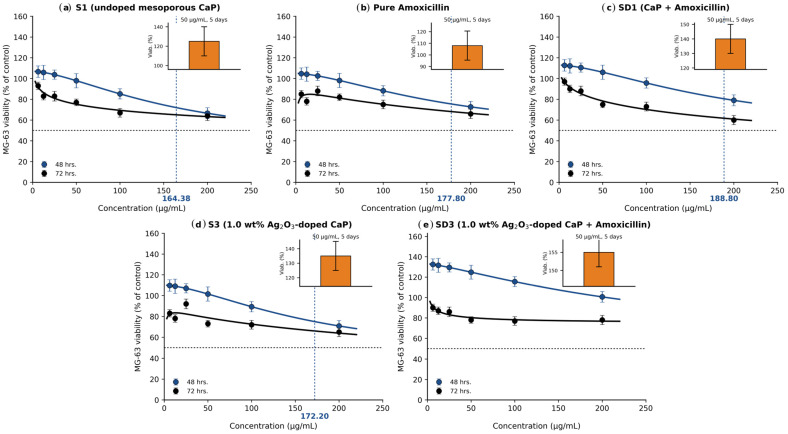
MTT cytocompatibility assay of MG-63 cells exposed to (**a**) S1 (undoped mesoporous CaP), (**b**) pure amoxicillin, (**c**) SD1 (undoped CaP loaded with amoxicillin), (**d**) S3 (1.0 wt% Ag_2_O_3_-doped CaP), and (**e**) SD3 (1.0 wt% Ag_2_O_3_-doped CaP loaded with amoxicillin). Main plots show MG-63 viability (% of control) as a function of concentration (µg/mL) at 48 h (blue) and 72 h (black); data are mean ± SD. Dashed horizontal lines indicate 50% viability; dashed vertical lines and adjacent values indicate the IC_50_ at 48 h, determined by nonlinear regression (four-parameter logistic fit) using software GraphPad Prism; no IC_50_ could be defined for SD3 within the tested concentration range. Inset bar plots show MG-63 viability (% of control) after 5 days (120 h) of exposure to 50 µg/mL of each formulation, corresponding to the proliferative response discussed in the Results.

The cytocompatibility of selected materials, namely pure mesoporous CaP (S1), pure amoxicillin, CaP loaded with amoxicillin (SD1), CaP doped with 1 wt% Ag_2_O_3_ (S3), and CaP doped with 1 wt% Ag_2_O_3_ and loaded with amoxicillin (SD3), was evaluated against MG-63 cells at 48, 72, and 120 h using the MTT assay. At 48 h, all samples with the exception of SD3 exhibited moderate cytotoxicity (Figure 9). Samples S1, S3, SD1, and pure amoxicillin yielded comparable IC_50_ values of 164, 172.2, 188.8, and 177.8 µg/mL, respectively (Table 5). Despite displaying approximately 40% cytotoxicity at the highest tested concentration, no IC_50_ values were detected for any of the samples after 72 h (Figure 9).

**Figure 10:**
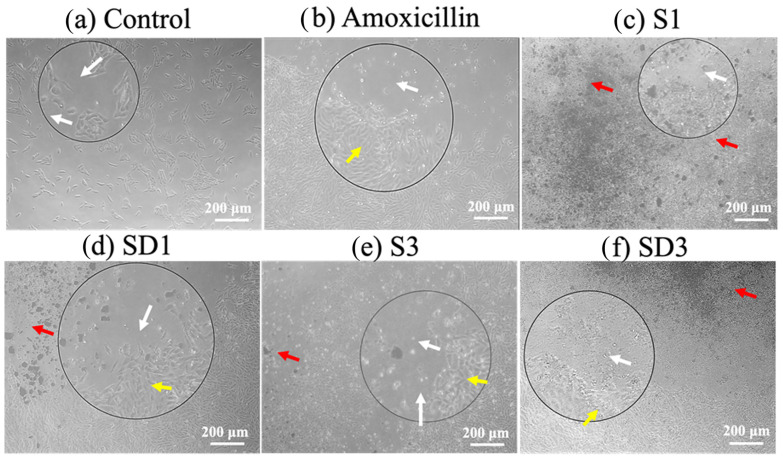
Phase-contrast light micrographs of MG-63 cells after 5 days (120 h) of exposure to control conditions and to each tested formulation at 50 µg/mL. White arrows indicate cell-free zones, areas of incomplete cell sheet formation in the control, and regions of impaired cell attachment immediately surrounding the deposited sample material in the treated groups. Yellow arrows indicate regions of confluent, highly proliferative cell sheets distal to the sample deposits. Red arrows indicate the deposited sample material itself, visible as dark aggregated particles. Circular insets show magnified views of representative regions illustrating cell morphology and sheet continuity for each sample. Scale bar = 200 µm.

Figure 10 illustrates low and homogeneous cell proliferation in the control cells. Following sample addition, the cells exhibited a tendency to migrate away from the bulk samples, displaying high confluency in regions distal to the sample deposits.

**Figure 11:**
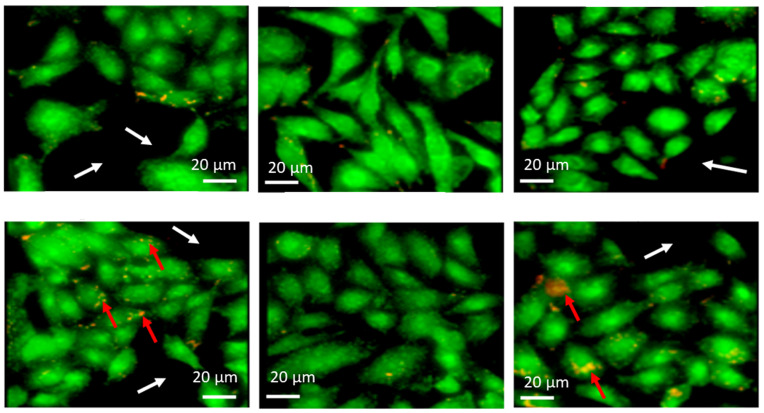
Fluorescence micrographs of MG-63 cells after 5 days (120 h) of exposure to each tested formulation at 50 µg/mL, stained with acridine orange/ethidium bromide (AO/EtBr) to assess cell membrane integrity and death mode. Viable, proliferating cells display intact membranes with uniform green fluorescence. White arrows indicate cell-free areas, corresponding either to regions of low proliferation in the control or to zones distal from/unaffected by the deposited sample material. Red arrows indicate apoptotic bodies, identified by condensed/fragmented chromatin and orange-red fluorescence. Overall, the micrographs revealed well-preserved cell morphology with intact cellular membranes and uncompromised nuclei devoid of shrinkage. The focal appearance of apoptotic bodies in the treated cells is noteworthy, particularly in the CaP samples loaded with amoxicillin (SD1 and SD3). With respect to the assessment of cellular death mechanisms, AO labelled both viable and non-viable cells, while EtBr selectively stained cells with compromised membrane integrity. Consequently, actively proliferating viable cells displayed green fluorescence, early apoptotic cells exhibited green colouration interspersed with yellow flecks, reflecting chromatin condensation and nuclear fragmentation, while necrotic cells appeared dark orange to red. The results indicated that the cytotoxic activity of the samples was contingent upon both incubation time and sample composition. The bioactive species released from the samples promoted substantial cell proliferation; however, this effect was attenuated in the presence of large, non-soluble particulates. Over time, the fabricated mesoporous nanopowders incorporating amoxicillin demonstrated the capacity to support MG-63 cell proliferation and viability. As corroborated by fluorescence imaging, the cells exhibited well-preserved morphology, intact membranes, and non-condensed, non-pyknotic nuclei devoid of chromatin condensation. Collectively, these findings highlight the potential of the developed nanopowders as platforms for drug delivery and bone tissue regeneration. These results are consistent with prior studies on hydroxyapatite-based materials, which are well recognized for their bioactive properties and compatibility with osseous tissue [74,75]. Furthermore, HA has been reported to selectively suppress the growth of MG-63 cells while modestly promoting osteoblast proliferation [75]. The time-dependent increase in cell viability observed in this study can be mechanistically attributed to the osteoconductive nature of the CaP matrix and the bioactive ions it releases into the culture medium. As the mesoporous CaP degrades, it liberates Ca^2+^ and PO_4_^3−^ ions, which are known to be actively taken up by cells and to promote osteogenic commitment and proliferation of osteoblast-lineage cells, in part through calcium-channel-mediated signaling and phosphate-dependent metabolic pathways that support ATP synthesis and osteogenic gene expression [76]. This ion-mediated stimulatory effect is consistent with the broader recognition that the osteoconductivity of CaP biomaterials arises from their capacity to modulate the local ionic microenvironment in a manner favorable to bone-forming cells [77]. With respect to silver, the low silver content released from the Ag_2_O_3_-doped samples (S3, SD3) may additionally contribute to the proliferative response observed at later time points. While silver is well recognized for its antibacterial activity at higher concentrations, several studies report that low, sub-cytotoxic concentrations of silver ions can be well tolerated by osteoblast-lineage cells and, in some cases, support osteogenic maturation, whereas cytotoxic and anti-proliferative effects predominate only at higher doses [78]. This dose-dependent behavior is consistent with the present findings, in which SD3 exhibited comparable cytotoxicity to the other formulations at 48 h but the highest proliferative response (155%) by 120 h, suggesting that the silver released from this formulation remained within a range compatible with, and possibly supportive of, sustained cell growth rather than inducing persistent toxicity. Taken together, these observations support a mechanism whereby the initial, modest cytotoxicity observed at 48 h reflects a transient response to burst-released ionic species and residual synthesis by-products, while the subsequent decline in cytotoxicity and rise in proliferation reflect progressive equilibration of the local ionic environment, continued osteoconductive stimulation by Ca^2+^/PO_4_^3−^, and in the silver-containing formulations a shift toward sub-toxic, potentially osteogenically favorable Ag^+^ concentrations as release proceeds.

White arrows indicate cell-free areas, red arrows indicate the samples, and yellow arrows indicate regions of highly confluent cells. The scale bar is 200 µm. After 5 days of treatment, the cytotoxic effect was attenuated, and a proliferative response became apparent, with increased cell growth of 125%, 135%, 140%, and 155% observed for S1, S3, SD1, and SD3, respectively. These findings were corroborated by the cell viability data presented in Figure 10 and Figure 11. In general, MG-63 osteosarcoma cells derived from human bone exhibit distinct morphological characteristics compared to osteoblasts, presenting as oval- to spindle-shaped cells without branching processes. Control cells were sub-confluent, occupying no more than 50–60% of the available growth area. In contrast, all treated cells displayed a cell-free zone surrounding the site of sample deposition, indicative of impaired cell-sample attachment. Cells located distal to the samples demonstrated markedly enhanced proliferation, with growth reaching up to 150% in the SD3 group. The fluorescence dye (AO/EtBr) findings shown in Figure 11 corroborated the observations obtained by contrast light microscopy in Figure 10.

While MG-63 cells provide a useful and widely used preliminary model for osteoblast-like behavior, they are derived from an osteosarcoma and may not fully recapitulate the phenotype and functional responses of normal bone cells; therefore, further validation using primary osteoblasts or mesenchymal stem cells is warranted to confirm the biocompatibility and osteogenic potential of the developed formulations. The physicochemical and morphological properties of the nanopowders further augment antibacterial efficacy through the capacity of metal oxide nanoparticles to scavenge reactive oxygen species. The sustained release of amoxicillin, governed by the microstructural characteristics of the nanopowders, is of particular relevance in the management of chronic osteomyelitis during bone healing. As such, these materials represent promising candidates for biomedical applications, effectively integrating the ability to promote healthy cell proliferation with potent antibacterial activity.

## 4. Conclusions

Silver-doped calcium phosphate loaded with amoxicillin was successfully synthesized via the sacrificial polymer template approach and characterized using several analytical techniques. The findings indicated that the incorporation of silver into mesoporous CaP nanopowders confers potential utility as a biomaterial, combining enhanced biocompatibility and antibacterial properties. The drug loading and delivery capacity of mesoporous CaP was confirmed to attain up to 46% cumulative release in phosphate-buffered saline (PBS). The presence of silver and amoxicillin synergistically enhanced the antibacterial efficacy against all investigated microorganisms. Cell viability and proliferation were found to be strongly dependent on the combined presence of silver and amoxicillin, with an optimal silver concentration of 1.0 wt% identified. The results collectively demonstrate that the prepared mesoporous CaP, both undoped and silver-doped, exhibited robust antibacterial activity as well as significant promotion of cell proliferation, underscoring its applicability as a platform for drug delivery and bone tissue engineering.

## Figures and Tables

**Table 1:** Nominal composition (wt%) of prepared samples (calculated from precursor masses weighed prior to synthesis).

Sample	Cap (wt%)	Ag_2_O_3_ (wt%)	Amoxicillin (wt%)
S1	100.0	0.0	0.0
S2	99.5	0.5	0.0
S3	99.0	1.0	0.0
SD1	100.0	0.0	10.0
SD2	99.5	0.5	10.0
SD3	99.0	1.0	10.0

**Table 2:** BET surface area metrics of the prepared samples.

Sample	Surface Area (m^2^/g)	Mean Pore Volume (cc/g)	Mean Pore Radius (nm)
S1	24.362	4.54	14.61
S2	12.2832	3.95	17.53
S3	10.5634	3.37	14.7

**Table 3:** Release kinetic parameters determined by model fitting.

Sample	Zero-Order R^2^	Higuchi R^2^	Korsmeyer–Peppas R^2^	t50 (h)	t90 (h)	nnn	RE 0–672 h (%)
SD1	0.884	0.988	0.148	760.2322	270.7580	0.4058	4.5986
SD2	0.905	0.991	0.979	126.0306	434.4375	0.3047	3.6896
SD3	0.928	0.992	0.983	23.5657	789.5927	0.3036	2.8433

**Table 4:** Inhibition zone diameter (mm) of test samples (mean ± SD, n = 3).

Microorganism	S1	S2	S3	SD1	SD2	SD3	Reference (CN/MCZ)
*Escherichia coli*	10.0 ± 0.50	12.0 ± 0.71	15.0 ± 0.92	15.0 ± 0.89	12.0 ± 0.60	16.5 ± 0.94	CN/MCZ (10 µg)
*Staphylococcus aureus*	9.5 ± 0.64	12.0 ± 0.83	15.0 ± 0.86	16.0 ± 0.85	13.0 ± 0.68	17.0 ± 0.87	CN/MCZ (10 µg)
*Candida albicans*	9.8 ± 0.56	11.0 ± 0.75	14.0 ± 0.97	13.0 ± 0.76	11.0 ± 75	16.0 ± 0.85	CN/MCZ (10 µg)

CN: Gentamicin (10 µg) disc. MCZ: Miconazole (10 µg) disc. Data are presented as mean ± SD (n = 3 independent experiments; each inhibition zone measured in triplicate).

**Table 5:** IC_50_ values on MG63 cell line at different time points (Units: µg/mL).

Sample	48 h	72 h
S1	164.3 ± 10.5	ND
SD1	172.2 ± 5.5	ND
S3	188.8 ± 15.4	ND
SD3	ND	ND
Amoxicillin	177.8 ± 14.2	ND

ND = Not detected.

## Data Availability

Data will be made available upon request.
